# Treatment of penetrating injuries of the retrohepatic vena cava: systematic review protocol

**DOI:** 10.1590/1677-5449.202400032

**Published:** 2024-10-09

**Authors:** Adenauer Marinho de Oliveira Góes, Simone de Campos Vieira Abib, Gustavo Henrique Dumont Kleinsorge, Daniella Adrea Araujo Rossi Vieira, Luis Carlos Uta Nakano

**Affiliations:** 1 Faculdade de Medicina da Universidade Federal do Pará – UFPA, Belém, PA, Brasil.; 2 Universidade Federal de São Paulo – UNIFESP, São Paulo, SP, Brasil.; 3 Hospital João XXIII, Fundação Hospitalar do Estado de Minas Gerais – FHEMIG, Belo Horizonte, MG, Brasil.

**Keywords:** inferior vena cava, wounds and injuries, endovascular procedures, therapeutics, surgical procedures, operative, systematic review

## Abstract

Injuries to the retrohepatic segment of the inferior vena cava require complex procedures, as exposure without prior vascular control can lead to uncontrollable and fatal bleeding. To achieve such control, the classic techniques of hepatic vascular exclusion and the implantation of an atriocaval shunt have been described, and more recently, endovascular strategies have been reported. However, there is no consensus in the literature regarding which of these strategies is associated with lower mortality. In order to determine which therapeutic strategy presents the lowest mortality and complication rates in the treatment of penetrating injuries to the retrohepatic segment of the inferior vena cava, a systematic review of the literature will be conducted, registered on the PROSPERO platform under the number CRD42023464133. The Cochrane Handbook for Systematic Reviews of Interventions will guide the process. Searches will be carried out in the MEDLINE/PubMed, LILACS, Embase, Scopus, and Web of Science databases. ClinicalTrials.gov and the International Clinical Trials Registry Platform (ICTRP) will be consulted to detect ongoing or unpublished trials. Studies will be selected based on a predefined search strategy, the number of results will be filtered using the Rayyan app, and the studies included will be independently reviewed by two authors to reach a final consensus. The qualitative analysis of the studies will be conducted using the RoB 1.0 tool.

## INTRODUCTION

The retrohepatic segment of the inferior vena cava (IVC) is contained in a closed compartment, delimited by the hepatic ligaments, the retroperitoneal space, the diaphragm, and the liver parenchyma.^1,2^ Exposure of this portion of the IVC involves complex surgical access, which may not be limited to laparotomy, but can demand varying types of thoracotomy access.^3,4^

Luckily, fewer than 10% of penetrating IVC injuries involve this segment. However, when it is involved, mortality can reach 90%.^5,6^ The time taken to control bleeding is one of the most important factors related to prognosis, demanding rapid pre-hospital care and surgical expertise in the many different techniques available to treat these injuries.^7-9^

In order to access the injured segment, it is often necessary to section the hepatic ligaments and mobilize the liver, exposing the cava.^10^ However, the liver parenchyma may be exerting partial tamponage on the injury, and attempts to achieve exposure without prior vascular control can often increase bleeding, which may become uncontrollable.^11,12^ Maneuvers such as triple hepatic vascular exclusion and atriocaval shunt placement have been described as methods to achieve prior vascular isolation of the injured segment.^13^

Triple hepatic exclusion (the Heaney maneuver) combines occlusion of hepatic hilum elements (the Pringle maneuver) with clamping of the infrahepatic and suprahepatic portions of the IVC, reducing bleeding via the wound when the liver is moved.^14^ However, since 2/3 of cardiac output is dependent on venous return via the IVC, abrupt reduction can provoke circulatory collapse in an already hypovolemic patient.^13-15^

Atriocaval shunt placement has been described with the objective of maintaining venous return from the IVC to the right atrium. This technique involves introduction of a tube into the right atrial appendage, positioning the other extremity in the suprarenal segment of the IVC, with cerclage at the intrapericardial IVC and above the renal veins, using Rumel tourniquets.^16-18^

The literature is replete with arguments in favor of and against both techniques and some case series report that both triple hepatic exclusion and atriocaval shunting are associated with survival of around 30%.^19,20^

The first report of use of resuscitative endovascular balloon occlusion of the aorta (REBOA) for cases of hemorrhagic shock was published in 1954.^21,22^ The technique was later adapted for provision of temporary hemostasis in IVC trauma while surgical access to the lesion is obtained to perform definitive hemostasis, giving rise to hybrid strategies for treating retrohepatic IVC injuries.^23-25^

Advances in materials and increasing experience with endovascular procedures, such as for treatment of aortic aneurysms, have made it possible to treat some cases of penetrating IVC traumas exclusively with endovascular procedures, such as with placement of covered stents, even in unstable patients with retrohepatic injuries.^26-28^ The advantages of this type of approach include reduced duration of surgery and bleeding and reduced organic response to trauma.^29^ However, use of endovascular intervention is often described for correction of iatrogenic injuries that happen in the hospital setting, reducing the time to treatment. In other words, under conditions that are often not comparable to those encountered when treating the victims of gunshot and knife wounds. Notwithstanding, endovascular repair in victims of non-iatrogenic injuries has been reported with increasing frequency.^30^ Despite all of the advances that have been achieved, the literature still has not reached consensus on which therapeutic strategy is best for treating penetrating injuries to the retrohepatic segment of the IVC.

## OBJECTIVES

To determine which technique achieves the lowest mortality and incidence of complications for treatment of penetrating injuries of the retrohepatic segment of the inferior vena cava in adult patients operated on an emergency basis.

## METHODS

This review protocol has been registered on the PROSPERO platform, under registration number CRD42023464133.

### Eligibility criteria

#### Types of studies

The review process will be guided by the Cochrane Handbook for Systematic Reviews of Interventions^31^ and will include parallel-group randomized controlled trials (RCTs), cluster-RCTs, and quasi-RCTs.

Non-randomized studies of interventions (NRSI) that analyze at least two comparison groups of interest will be included if RCTs and quasi-RCTs do not provide sufficient evidence. Retrospective observational studies, including cases series, will be included if the evidence from RCTs, quasi-RCTs, and prospective NRSIs is insufficient.

Any study that describes cases of penetrating injuries of the retrohepatic segment of the inferior vena cava in adult patients treated by triple hepatic exclusion, atriocaval shunting, or endovascular/hybrid techniques operated as an emergency will be considered.

#### Types of participants

Patients will be included of both sexes, aged 18 years or older, with penetrating injuries of the retrohepatic segment of the inferior vena cava confirmed by any type of imaging exam or by surgical exploration.

#### Types of interventions

Triple hepatic exclusion, atriocaval shunting, or use of endovascular/hybrid techniques will be considered as interventions for treatment of penetrating injuries of the retrohepatic segment of the IVC. Since there is no standard intervention established, the review will consider all possible comparisons between the interventions of interest.

### Sources of information

#### Search methods for identification of studies

The LILACS, Web of Science, MEDLINE/PubMed, Scopus, and Embase databases will be consulted. The preliminary MEDLINE search strategy will be adapted for use with the other databases and no filters will be applied (Table 1). The studies thus identified will be selected manually. Ongoing or unpublished studies will be consulted on the ClinicalTrials.gov platform and the International Clinical Trials Registry Platform (ICTRP), via the World Health Organization (WHO) portal.

**Table 1:** Draft search strategy (MEDLINE via PubMed).

#	Question	Results
#1	((vena cava[Title/Abstract]) AND (trauma OR injuries)[Title/Abstract])	4,209
#2	((inferior vena cava[Title/Abstract]) AND (trauma OR injuries)[Title/Abstract])	2,672
#3	(retro hepatic[Title/Abstract]) AND (cava[Title/Abstract])	58
#4	atrio-caval[Title/Abstract] AND shunt [Title/Abstract]	4

Databases will be searched from inception to the present, with no restrictions on publication language or status. Help from native speakers of languages that the authors are not familiar with can be requested via Cochrane Task Exchange (taskexchange.cochrane.org). Only the adverse effects described in the included studies will be considered.

#### Study selection

Titles and abstracts of all potential studies identified in the search results will be independently assessed by two reviewers. They will code each study as “included” (eligible or potentially eligible/unclear) or “excluded”, using the Rayyan tool.^32^ The full text study reports/publications will be accessed and both reviewers will assess them independently for inclusion and record the reasons for excluding ineligible studies.

Duplicates will be identified and excluded and multiple reports from a single study will be grouped so the study, rather than each report, is the unit of interest for the review. The selection process will yield sufficient detail to fill out a PRISMA 2020 flow diagram^33^ and a “Characteristics of excluded studies” table will be constructed. Studies will be reported as “full text”, as published as “abstract only”, or as “unpublished data”. Abstracts and conference proceedings will be considered eligible if they provide usable data.

Any disagreements during this process will be resolved by discussion or, if necessary, by arbitration by a third of the review authors.

#### Data extraction and management

The data extraction form will be tested by two reviewers, who will make any changes that are appropriate. Two reviewers will extract data from each study independently and in duplicate.

We will extract the following data from each study:

- Study design
- Method of analysis
- Outcome measures
- Duration of follow-up
- Numbers of participants at baseline and follow-up
- Type of population
- Percentage (%) per sex
- Mean age (standard deviation [SD])
- Covariates adjusted for
- Intervention employed
- Risk of bias, according to RoB 1.0
- Data used to calculate differences in clinical outcomes in the results of the interventions: percentage survival; use of blood products; length stay in the intensive care unit; need for hemodialysis
- Sources of study finance and authors’ declarations of interests

## RESULTS AND PRIORITIZATION

### Primary outcomes

Intrahospital all causes mortality: mortality will be analyzed as a dichotomous variable. We will not analyze the time before occurrence of death, regardless of cause.

### Secondary outcomes

Use of blood products: when reported, will be quantified according to the number of units of blood products employed, including packed red blood cells, platelets, and plasma.

Length of stay in the intensive care unit: when reported, will be quantified in days. Need for hemodialysis: assessed as a dichotomous variable. We will not analyze duration of renal substitution therapy.

## CONCLUSIONS

The review conclusions will be based on the synthesis of findings or a narrative of the studies included in the review. The review will be conducted in such a manner as to attempt to facilitate future evidence-based decision making on which therapeutic strategy to use to treat adult patients who have been the victims of penetrating injuries to the retrohepatic segment of the inferior vena cava.
