# Supplementary material for: Treatment of penetrating injuries of the retrohepatic vena cava: systematic review protocol
Source: J Vasc Bras. 2024 Oct 9;23:e20240003. doi: 10.1590/1677-5449.202400032 (PMC11486465; doi:10.1590/1677-5449.202400032)
Supplement: Translation

# Translation: Tratamento de lesões penetrantes de veia cava retro-hepática: protocolo de revisão sistemática

**DOI:** 10.1590/1677-5449.202400031

**Authors:** Adenauer Marinho de Oliveira Góes, Simone de Campos Vieira Abib, Gustavo Henrique Dumont Kleinsorge, Daniella Adrea Araujo Rossi Vieira, Luis Carlos Uta Nakano

**Affiliations:** 1 Faculdade de Medicina da Universidade Federal do Pará – UFPA, Belém, PA, Brasil.; 2 Universidade Federal de São Paulo – UNIFESP, São Paulo, SP, Brasil.; 3 Hospital João XXIII, Fundação Hospitalar do Estado de Minas Gerais – FHEMIG, Belo Horizonte, MG, Brasil.

**Keywords:** veia cava inferior, ferimentos e lesões, procedimentos endovasculares, procedimentos de tratamento, procedimentos cirúrgicos operatórios, revisão sistemática

## Abstract

Lesões do segmento retro-hepático da veia cava inferior requerem procedimentos complexos, pois sua exposição sem controle vascular prévio pode acarretar sangramentos incoercíveis e letais. Para este controle, a tríplice exclusão vascular hepática e o implante de *shunt* átrio-caval foram classicamente descritos e, mais recentemente, estratégias endovasculares foram relatadas. Entretanto, não há consenso na literatura sobre qual dessas estratégias apresenta menor mortalidade. Com a finalidade de definir qual estratégia terapêutica apresenta menor mortalidade e incidência de complicações no tratamento de lesões penetrantes do segmento retro-hepático da cava inferior, será realizada uma revisão sistemática da literatura, a qual foi registrada na plataforma PROSPERO sob o número CRD42023464133. O Cochrane Handbook for Systematic Reviews of Interventions será utilizado para orientar o processo. As buscas serão realizadas nas bases de dados MEDLINE/PubMed, LILACS, Embase, Scopus e Web of Science. O site ClinicalTrials.gov e a Plataforma Internacional de Registro de Ensaios Clínicos (ICTRP) serão consultados para a detecção de ensaios em andamento ou não publicados. Os estudos serão selecionados por uma estratégia de busca previamente estabelecida, o montante de resultados será filtrado utilizando-se o aplicativo Rayyan, e os estudos incluídos serão analisados independentemente por dois autores para construção do senso final. A análise qualitativa dos estudos será feita com a ferramenta RoB 1.0.

## INTRODUÇÃO

O segmento retro-hepático da veia cava inferior (VCI) está contido em um compartimento fechado, delimitado pelos ligamentos hepáticos, o retroperitônio, o diafragma e o parênquima hepático^1,2^ . A exposição dessa porção da VCI exige acessos cirúrgicos complexos, que podem não apenas requerer laparotomia, mas também diferentes acessos por toracotomias^3,4^ .

Felizmente, menos de 10% das lesões penetrantes de VCI acometem esse segmento. Entretanto, quando acometido, a mortalidade pode atingir 90%^5,6^ . O tempo até o controle da hemorragia é um dos principais fatores relacionados ao prognóstico, exigindo um atendimento pré-hospitalar rápido e *expertise* do cirurgião quanto às diversas técnicas disponíveis para tratamento desses ferimentos^7-9^ .

Para acessar o segmento lesionado, os ligamentos hepáticos frequentemente precisam ser seccionados e o fígado mobilizado, expondo a cava^10^ . Entretanto, o parênquima hepático pode promover tamponamento parcial da lesão, e a tentativa de exposição sem o prévio controle vascular frequentemente aumenta o sangramento, podendo tornar-se incoercível^11,12^ . Para o prévio isolamento vascular do segmento lesionado, foram descritas manobras como a tríplice exclusão hepática e o implante de *shunt* átrio-caval^13^ .

A tríplice exclusão hepática (manobra de Heaney) associa a oclusão dos elementos do hilo hepático (manobra de Pringle) ao pinçamento da VCI em suas porções infra-hepática e supra-hepática, diminuindo o sangramento pela lesão ao mobilizar o fígado^14^ . Entretanto, como 2/3 do débito cardíaco dependem do retorno venoso pela VCI, sua redução abrupta pode acarretar colapso circulatório em um paciente já hipovolêmico^13-15^ .

Objetivando manter o retorno venoso da VCI até o átrio direito, foi descrito o implante de um *shunt* átrio-caval. A técnica envolve a introdução de um tubo através do apêndice atrial direito, posicionando a outra extremidade no segmento suprarrenal da VCI, e a realização de uma cerclagem da VCI intrapericárdica e acima das veias renais, com torniquetes de Rumel^16-18^ .

A literatura está repleta de argumentos a favor e contra ambas as técnicas, e algumas séries de casos descrevem que tanto a tríplice exclusão hepática quanto o implante do *shunt* átrio-caval proporcionam sobrevida em torno de 30%^19,20^ .

O primeiro relato do uso de balão endovascular para oclusão de aorta (REBOA) objetivando ressuscitação em casos de choque hemorrágico foi em 1954^21,22^ . A técnica foi posteriormente adaptada para hemostasia temporária em traumas de VCI, enquanto o acesso cirúrgico à lesão era executado para hemostasia definitiva, dando origem a estratégias híbridas para tratamento de lesões de VCI retro-hepática^23-25^.

O desenvolvimento de materiais e o acúmulo de experiência em procedimentos endovasculares, como o tratamento de aneurismas da aorta, possibilitou que alguns casos de ferimentos penetrantes da VCI, incluindo os de pacientes instáveis com lesão retro-hepática, fossem tratados exclusivamente com procedimentos endovasculares, como o implante de *stents* recobertos^26-28^ . Entre as vantagens desse tipo de abordagem, são citadas a redução do tempo cirúrgico e do sangramento e a diminuição da resposta orgânica ao trauma^29^ . Entretanto, a abordagem endovascular é frequentemente descrita para a correção de lesões iatrogênicas que ocorreram já em ambiente hospitalar, reduzindo o tempo até o tratamento. Ou seja, condições que frequentemente não são comparáveis àquelas encontradas nas vítimas de ferimentos por armas de fogo e armas brancas. Mesmo assim, reparos endovasculares em vítimas de lesões não iatrogênicas de VCI vêm sendo relatados com frequência crescente^30^ . Apesar de todos os avanços, a literatura ainda não apresenta um consenso sobre qual a melhor estratégia terapêutica para o tratamento das lesões penetrantes do segmento retro-hepático da VCI.

## OBJETIVOS

Definir qual técnica apresenta menor mortalidade e incidência de complicações para o tratamento de lesões penetrantes do segmento retro-hepático de veia cava inferior em pacientes adultos operados em caráter de emergências.

## MÉTODOS

O presente protocolo de revisão foi registrado na plataforma PROSPERO sob o número CRD42023464133.

### Critérios de elegibilidade

#### Tipos de estudos

O processo de revisão será orientado pelo Cochrane Handbook for Systematic Reviews of Interventions^31^ , considerando-se estudos de ensaios clínicos controlados randomizados (ECRs) paralelos, *clusters* e quase ECRs.

Estudos não randomizados (ENR) de intervenções, desde que considerem ao menos dois grupos comparativos de interesse, serão incluídos caso os ECRs e quase ECRs não forneçam evidência suficiente. Estudos observacionais retrospectivos, incluindo séries de casos, serão incluídos no caso de evidência insuficiente fornecida por ECRs, quase ECRs e ENR prospectivos.

Qualquer estudo que descreva casos de lesões penetrantes do segmento retro-hepático da veia cava inferior em pacientes adultos tratados por tríplice exclusão hepática, *shunt* átrio-caval ou técnicas endovasculares/híbridas operados em caráter de emergência, será considerado.

#### Tipos de participantes

Serão incluídos pacientes de ambos os sexos, com idade igual ou superior a 18 anos, com lesões penetrantes do segmento retro-hepático da veia cava inferior confirmada por qualquer exame de imagem ou por exploração cirúrgica.

#### Tipos de intervenções

Serão consideradas como intervenções para o tratamento de lesões penetrantes do segmento retro-hepático da VCI a tríplice exclusão hepática, o implante de *shunt* átrio-caval ou o uso de técnicas endovasculares/híbridas. Como não há uma intervenção padrão estabelecida, serão consideradas todas as comparações possíveis entre as intervenções de interesse.

### Fontes de informações

#### Métodos de busca para identificação de estudos

As bases de dados LILACS, Web of Science, MEDLINE/PubMed, Scopus e Embase serão consultadas. A estratégia de busca preliminar do MEDLINE será adaptada para uso em outras bases de dados e não será aplicado nenhum filtro (Tabela 1). Os estudos identificados serão selecionados manualmente. Ensaios em andamento ou não publicados serão pesquisados nas plataformas ClinicalTrials.gov e na Plataforma Internacional de Registro de Ensaios Clínicos (ICTRP) por meio do portal da Organização Mundial de Saúde (OMS).

**Tabela 1:** Rascunho da estratégia de pesquisa (MEDLINE via PubMed).

#	Question	Resultado
#1	((vena cava[Title/Abstract]) AND (trauma OR injuries)[Title/Abstract])	4.209
#2	((inferior vena cava[Title/Abstract]) AND (trauma OR injuries)[Title/Abstract])	2.672
#3	(retro hepatic[Title/Abstract]) AND (cava[Title/Abstract])	58
#4	atrio-caval[Title/Abstract] AND shunt [Title/Abstract]	4

Os bancos de dados serão pesquisados desde o início dos registros até o presente, sem restrição quanto ao idioma ou o status da publicação. O auxílio de falantes nativos de línguas específicas, quando não familiares aos autores, poderá ser solicitado através da Cochrane Task Exchange (taskexchange.cochrane.org). Apenas os efeitos adversos descritos nos estudos incluídos serão considerados.

#### Seleção de estudos

Títulos e resumos de todos os estudos potenciais identificados como resultado da pesquisa serão independentemente avaliados por dois revisores. Então, eles codificarão esses estudos como “incluído” (elegível ou potencialmente elegível/não claro) ou “excluído”, utilizando a ferramenta Rayyan^32^ . Relatórios/publicações de estudo em texto completo serão recuperados e dois revisores os examinarão independentemente para inclusão, além de registrar as razões para a exclusão dos estudos inelegíveis.

Duplicatas serão identificadas e excluídas e relatórios de um mesmo estudo serão agrupados para que cada estudo, em vez de cada relatório, seja a unidade de interesse na revisão. O processo de seleção terá detalhes suficientes para preencher um diagrama de fluxo PRISMA 2020^33^ e a tabela “Características dos estudos excluídos” será elaborada. Serão considerados estudos relatados como “texto completo”, aqueles publicados apenas como “resumo” e também “dados não publicados”. Resumos e anais de conferências serão considerados como elegíveis se tiverem dados utilizáveis.

Quaisquer eventuais divergências durante o processo serão resolvidas por meio de discussão ou, se necessário, pela arbitragem de um terceiro autor desta revisão.

#### Extração e gerenciamento dos dados

O formulário de extração de dados será testado por dois revisores, que farão as alterações apropriadas. Dois revisores em duplicata extrairão os dados de cada estudo independentemente.

Extrairemos os seguintes dados de cada estudo:

- Desenho do estudo
- Método de análise
- Medidas de resultado
- Duração do acompanhamento
- Número de participantes na linha de base e acompanhamento
- Tipo de população
- Percentual (%) por gênero
- Idade média (desvio padrão [DP])
- Covariáveis ajustadas
- Intervenção utilizada
- Risco de viés com RoB 1.0
- Dados para calcular as diferenças quanto aos desfechos clínicos nos resultados das intervenções: porcentagem de sobrevida; uso de hemoderivados; tempo de internação em unidade de terapia intensiva; necessidade de hemodiálise
- Fontes de financiamento do estudo e declarações de interesses dos autores

## RESULTADOS E PRIORIZAÇÃO

### Desfechos primários

Mortalidade intra-hospitalar por todas as causas: a mortalidade será avaliada como variável dicotômica. Não consideraremos o tempo para ocorrência do óbito, independente das causas.

### Desfechos secundários

Uso de hemoderivados: quando for detectado, será quantificado de acordo com o número de unidades de hemoderivados utilizados, incluindo concentrado de hemácias, plaquetas e plasma.

Tempo de internação em unidade de terapia intensiva: quando for detectado, será quantificado em dias. Necessidade de hemodiálise: será avaliada como variável dicotômica. Não consideraremos o tempo de duração da terapia de substituição renal.

## CONCLUSÃO

A conclusão do trabalho será baseada na síntese dos achados ou narrativa dos trabalhos incluídos nesta revisão. Além disso, o trabalho será coordenado de forma a tentar tornar exequível uma futura tomada de decisão quanto a qual estratégia terapêutica usar, baseada em evidências, ao tratar pacientes adultos vítimas de lesão penetrante no segmento retro-hepático da veia cava inferior.
